# Antithrombin plus alpha-1 protease inhibitor does not affect coagulation and inflammation in two murine models of acute lung injury

**DOI:** 10.1186/s40635-019-0240-7

**Published:** 2019-07-25

**Authors:** Jenny Juschten, Sarah Anne Ingelse, Martinus Adrianus Wilhelmus Maas, Armand Roelof Johan Girbes, Nicole Petra Juffermans, Marcus Josephus Schultz, Pieter Roel Tuinman

**Affiliations:** 1 Department of Intensive Care, Amsterdam UMC, VU Medical Center, Amsterdam, Netherlands; 2 Research VUmc Intensive Care (REVIVE), Amsterdam UMC, VU Medical Center, Amsterdam, Netherlands; 3 Department of Intensive Care, Amsterdam UMC, Academic Medical Center, Amsterdam, Netherlands; 4 Laboratory of Experimental Intensive Care and Anesthesiology (L·E·I·C·A), Amsterdam UMC, Academic Medical Center, Amsterdam, Netherlands; 5 Emma Children’s Hospital—Pediatric Intensive Care Unit, Amsterdam UMC, Academic Medical Center, Amsterdam, Netherlands; 6 Mahidol Oxford Tropical Medicine Research Unit (MORU), Mahidol University, Bangkok, Thailand

**Keywords:** Antithrombin, Alpha-1 protease inhibitor, Acute lung injury, ARDS, Lung injury model, Inflammation, Coagulation

## Abstract

**Background:**

In acute respiratory distress syndrome (ARDS), uncontrolled production of activators of coagulation and proinflammatory mediators results in a shift from an adequate local innate immune response to hypercoagulability and inflammation. This study aimed to investigate whether the protease inhibitors antithrombin (AT) and alpha-1 protease inhibitor (A1PI) may attenuate an exaggerated pulmonary immune response.

**Methods:**

Lung injury was induced either by single intranasal administration of lipopolysaccharide (LPS) (5 mg/kg) in BALB/c mice or by combination of an intravenous injection of LPS (10 mg/kg) with subsequent injurious ventilation using high tidal volumes (12–15 ml/kg) for 4 h in RccHan Wistar rats. Animals received either a single bolus of AT (250 IU/kg) or A1PI (60 mg/kg) alone or in combination, with or without intravenous low-dose heparin (100 U/kg). Control animals received saline. Additional controls received neither LPS, nor ventilation, nor treatment. Endpoints were local and systemic markers of coagulation, e.g., thrombin–antithrombin complexes (TATc), and inflammation, e.g., interleukin-6.

**Results:**

Both lung injury models resulted in a pronounced immune response within the pulmonary compartment shown by elevated levels of markers of coagulation and inflammation. The two-hit lung injury model also induced profound systemic coagulopathy and inflammation. Monotherapy with AT or A1PI did not reduce pulmonary coagulopathy or inflammation in any lung injury model. Nor did combination therapy with AT and A1PI result in a decrease of coagulation or inflammatory parameters. AT markedly reduced systemic levels of TATc in the two-hit lung injury model. Systemic inflammation was not affected by the different interventions. Additional administration of heparin did not lead to macroscopic bleeding incidences.

**Conclusions:**

In two different murine models of acute lung injury, neither single therapy with AT or A1PI nor combination of both agents attenuates the pronounced pulmonary coagulation or inflammatory response.

## Background

ARDS is a heterogeneous syndrome characterized by local production of coagulation proteases and proinflammatory mediators causing a shift from a potentially adequate innate immune response to harmful coagulation and inflammation [1, 2]. In ARDS patients, the degree of pulmonary hypercoagulability and inflammation is clearly linked to clinical outcome [3–5].

Antithrombin (AT) and alpha-1 protease inhibitor (A1PI) are serine protease inhibitors playing a pivotal role in maintaining equilibrium between protective and harmful coagulation and inflammation [6–8]. AT contributes to a balanced coagulation response by inhibiting the activity of thrombin, a coagulation factor facilitating the conversion of fibrinogen to fibrin [9]. Preclinical studies also suggest an anti-inflammatory effect of AT by downregulating the inflammatory response and preventing premature neutrophil activation [10, 11]. A1PI is an acute-phase protein exerting anti-inflammatory effects by blocking proteolytic enzymes released by activated neutrophils into the pulmonary compartment, acting as an “anti-protease screen” [7]. Previously, both AT and A1PI were administered in an indirect ARDS model in sheep leading to attenuation of microvascular protein permeability within the lungs and prevention of a decrease in arterial oxygenation. None of these effects were observed when only one intervention was administered [12].

Studies in ARDS patients with anticoagulants, such as protein C, heparin, or AT, did not result in improved outcome despite inhibiting an exaggerated coagulation response [13–17]. As coagulation and inflammation are closely interrelated and act as “brothers-in-arms,” treatment strategies addressing both host-protective responses may be more successful in restoring a balanced immune response than one intervention alone. We hypothesized that combination therapy of AT or A1PI exerts a beneficial synergistic effect by decreasing pulmonary coagulopathy and inflammation, as well as improving endothelial dysfunction compared to AT or A1PI therapy alone. Therefore, we investigated the effect of combination therapy on pulmonary coagulation and inflammation in two models of acute lung injury. Heparin was added in an extra group receiving combination therapy to assess a potential increased bleeding risk.

## Methods

### Animals

The experiments were conducted under protocols approved by the Animal Care and Use Committee of the Academic Medical Center at the University of Amsterdam (LEICA132-AB and -AD). Animals were used in compliance with the Institutional Standards for Use of Laboratory Animals and were handled 1 week before experiments to diminish stress activation. They were housed in a specific-pathogen-free facility on a 12/12-h light/dark cycle. Standard laboratory chow and water were available ad libitum. *Single-hit lung injury model*: Experiments were performed in 44 male specific-pathogen-free BALB/c mice (Charles River, The Netherlands), aged 8–12 weeks and with a mean ± SD body weight of 23.4 ± 1 .2 g. *Two-hit lung injury model*: Experiments were performed in 45 male specific-pathogen-free RccHan Wistar rats (Envigo, The Netherlands) with a mean ± SD body weight of 322 ± 19 g.

### Study groups

Animals were randomized into five different intervention groups: (1) antithrombin (AT; 250 IU/kg; Anbinex™, kind gift from Grifols), (2) alpha-1 protease inhibitor (A1PI; 60 mg/kg; Prolastin™, kind gift from Grifols), (3) AT + A1PI, (4) AT + A1PI + heparin (100 U/kg; LEO Pharma, Lier, Belgium), and (5) sodium chloride 0.9% (saline; Braun, Melsungen, Germany), referred to as the “hit” group (*N* = 8). Control animals received neither LPS, nor ventilation, nor treatment (*N* = 4). Administered dosages of intervention drugs were based on previous preclinical research [18–20].

### Study design

#### Single-hit lung injury model

**Fig. 1:**
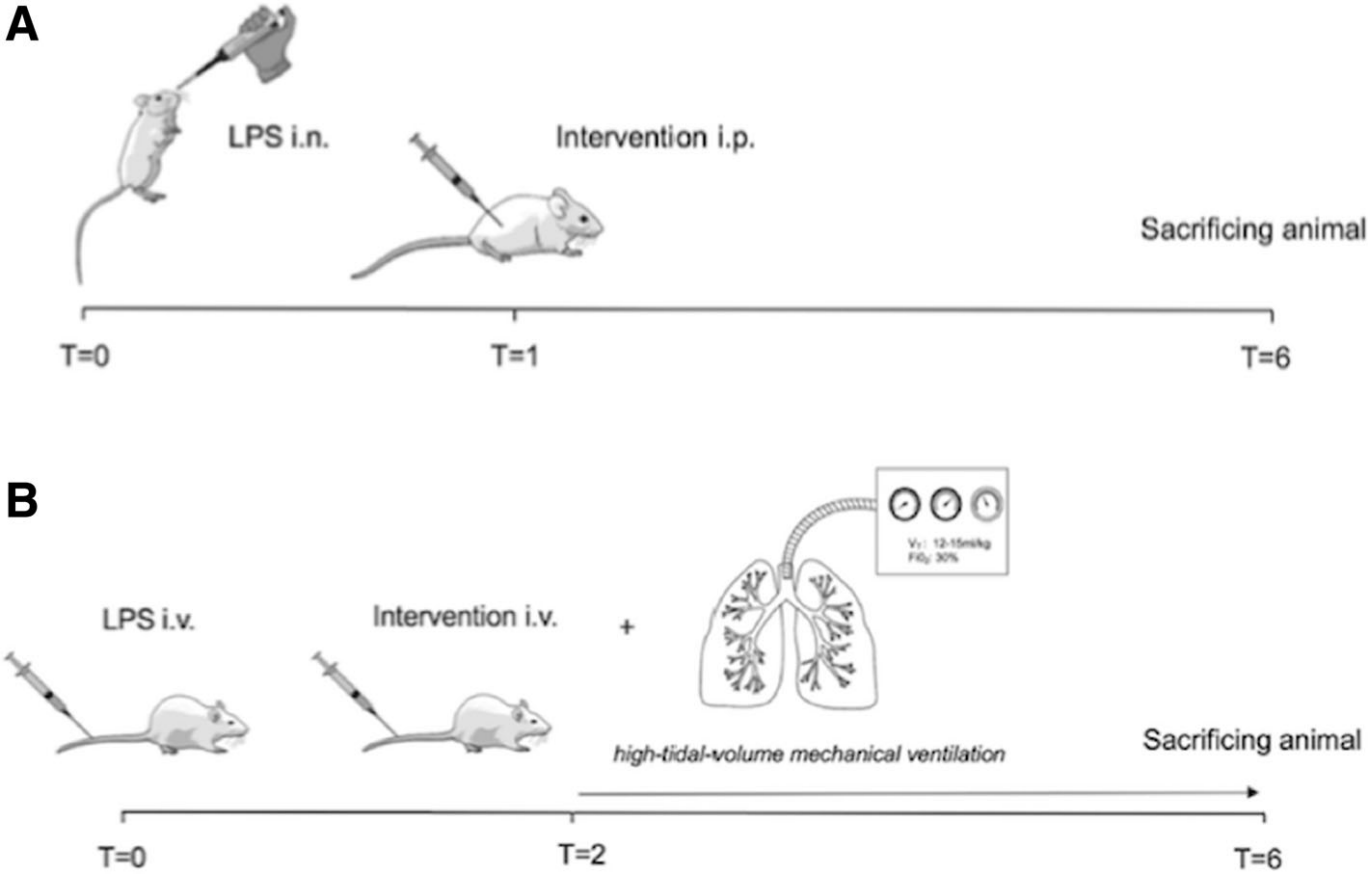
Lung injury models. **a** Single-hit lung injury model. **b** Two-hit lung injury model. Abbreviations: FiO_2_, fraction of inspired oxygen; i.n., intranasal; i.p., intraperitoneal; i.v., intravenous; LPS, lipopolysaccharide; *T* time point in hours; *V*_*T*_, tidal volume

At baseline, mice were weighed and labeled. At time point *T* = 0, mice were sedated using isoflurane 2–4%, followed by intranasal (i.n.) inoculation of 5 mg/kg LPS (*Escherichia coli*, serotype: 0127:B8, Sigma Aldrich, St. Louis, MO, USA) diluted in 50 μl NaCl 0.9% or 50 μl NaCl 0.9% (control). Appropriate dosages of LPS to achieve an adequate lung injury model were assessed in a prior pilot trial (data not shown). One hour after i.n. inoculation (*T* = 1), mice received the intervention drugs intraperitoneally (i.p.). Five hours after administration of the intervention (*T* = 6), mice were anesthetized using a bolus of 9.5 μl KDA per gram body weight i.p. (KDA 1.26 ml 100 mg/ml ketamine (Anesketin, EuroVetAnimal Health B.V., Bladel, The Netherlands) + 0.2 ml 0.5 mg/ml dexmedetomidine (Pfizer Animal Health B.V., Capelle a/d Ijssel, The Netherlands) + 1 ml 0.5 mg/ml atropine (Pharmachemie, Haarlem, The Netherlands) in 5 ml 0.9% NaCl) and sacrificed by exsanguination from the carotid artery (Fig. 1a). Whole blood was drawn using a heparin-coated syringe and subsequently centrifuged for 10 min at 4000 rpm at 4 °C (Eppendorf, microcentrifuge). Plasma was obtained and stored at − 80 °C for further analyses. Following exsanguination, the sternum of the mouse was opened surgically and lungs with bronchi and trachea were resected. To obtain bronchoalveolar lavage fluid (BALF), the right main bronchus was clipped and a 1-ml syringe was connected to the trachea. The left lung was lavaged three times with 0.5 ml normal saline. BALF was centrifuged for 10 min at 2000 rpm at 4 °C (Eppendorf, microcentrifuge); the upper lobe of the right lobe was snap frozen in liquid nitrogen and stored at − 80 °C for further analyses.

#### Two-hit lung injury model

Intravenous (i.v.) injection of LPS to mimic sepsis-induced ARDS was regarded as the first hit to the lungs, followed by high tidal volume mechanical ventilation for ventilator-induced lung injury (VILI) as the second pulmonary hit. At baseline (*T* = 0), rats were sedated using isoflurane 2–4%, followed by an i.v. injection of 10 mg/kg LPS (*Escherichia coli*, serotype: 0127:B8, Sigma Aldrich, St. Louis, MO, USA) diluted in 1 ml NaCl into the penile vein. Dosage of LPS to achieve an adequate sepsis-induced ARDS model was assessed in a pilot trial (data not shown). Simultaneously, rats received one dosage of 0.03 mg/kg buprenorphine (Temgesic®; Indivior UK Limited, Slough, UK) subcutaneously. Rats were placed back into their cage. Two hours after LPS injection (*T* = 2), rats were sedated by i.p. injection of 85 mg/kg pentobarbital (Euthasaol® 20%; AST Farma B.V., Oudewater, The Netherlands) and intervention drugs were administered intravenously via the tail vein. Tracheotomy was performed, and a polyethylene catheter was inserted into the carotid artery for hemodynamic monitoring and sampling for blood gas analysis (*T* = 2, *T* = 4, and *T* = 6). Anesthesia was maintained by continuous intravenous infusion of pentobarbital at a rate of 15 mg/kg/h, and fluid resuscitation was performed by infusing Ringer’s lactate (Baxter; Utrecht, The Netherlands) at a rate of 8 ml/kg/h to maintain a mean arterial pressure of above 60 mmHg. In case of hypotension, a maximum of three boluses of 10 ml/kg were administered. Body temperature was maintained between 36.5 and 37.5°C using a heat pad. Rats were subjected to a mechanical ventilation in a volume-controlled mode with tidal volumes between 12 and 15 ml/kg and a positive end-expiratory pressure (PEEP) of 3.4 mbar (Babylog® 8000, Dräger, Germany). The inspired oxygen fraction (Fi0_2_) was set at 30%, the respiratory rate at 60 breaths per minute with an inspiratory-to-expiratory ratio of 1:1.5. Rats were mechanically ventilated for 4 h and sacrificed by exsanguination from the carotid artery after a bolus of pentobarbital (*T* = 6) (Fig. 1b). Arterial blood was drawn using a heparin-coated syringe and subsequently centrifuged for 15 min at 1500 rcf at 4 °C (Eppendorf, Centrifuge 5804 R). Plasma was obtained and stored at − 80 °C for further analyses. Following exsanguination, the sternum of the rat was opened surgically and lungs with bronchi and trachea were resected. For BALF, the right main bronchus was clipped and the left lung was lavaged three times with 2 ml sterile saline. BALF was centrifuged for 15 min at 1500 rcf at 4 °C and stored at − 80 °C for further analyses. The middle and accessory lobe were snap frozen in nitrogen and stored at − 80 °C until processing to lung homogenate. Plasma, BALF, and lung homogenate samples were analyzed using a coding system; hence, the outcome assessor was blinded for which treatment each animal received.

### Coagulation and fibrinolysis

#### Single-hit lung injury model

Thrombin–antithrombin complex (TATc) levels were determined as a marker for coagulation and assessed in BALF and plasma using a mouse-specific ELISA kit (Abcam, Cambridge, The UK) according to the manufacturer’s instructions, detection range 0.031–8 ng/ml.

#### Two-hit lung injury model

Markers for coagulation and fibrinolysis, e.g., thrombin–antithrombin complexes (TATc) and plasminogen activator inhibitor (PAI)-1, were analyzed in BALF and plasma using rat-specific ELISA kits (Nordic Biosite AB, Täby, Sweden) according to the manufacturer’s instructions, detection range: TATc 15.625–1000 pg/ml, PAI-1 0.313–20 ng/ml.

### Inflammatory response and endothelial injury

#### Single-hit lung injury model

Inflammatory cytokines and endothelial markers, e.g., tumor necrosis factor (TNF)-α, interleukin (IL)-1β, were assessed in BALF, and TNFα, IL-1β, IL-6, and keratinocyte-derived chemokine (KC) were assessed in plasma using the multi-analyte bead assay Luminex (Bio-Techne, R&D systems, Minneapolis, MN, USA) according to the manufacturer’s instructions. Detection limits for each biomarker are TNFα 1.13, IL-1β 100.55, KC 24.02, and IL-6 18.84. Total protein levels in BALF were assessed using Bradford Protein Assay Kit (OZ Biosciences, Marseille, France) according to official instructions with bovine serum albumin (BSA) as standard and a detection limit of 50 μg/ml.

#### Two-hit lung injury model

Inflammatory cytokines, e.g., interleukin (IL)-6 and cytokine-induced neutrophil chemoattractant (CINC)-3, were assessed in BALF and plasma using rat-specific ELISA kits (R&D systems; Minneapolis, MN, USA) according to the manufacturer’s instructions. Detection limits for each biomarker are the following: IL-6 125–8000 pg/ml and CINC-3 31.2–2000 pg/ml. Myeloperoxidase (MPO) activity was measured in lung homogenate and plasma using a rat-specific ELISA kit according to the manufacturer’s instructions (Hycult Biotech; Uden, The Netherlands). Total protein levels in BALF were determined by the bovine serum albumin (BSA) Lowry method.

### Statistical analyses

A sample size of eight animals per group was needed, using a power of 0.8 with a chance of 0.904 (effect size 1.85) with double signification level of 0.05 to detect a difference in IL-6 levels between intervention groups and the control group. All data are expressed as median (interquartile range (IQR)) according to data distribution. The saline control and healthy control groups were compared using the Mann–Whitney *U* test. Comparisons between the intervention groups and the saline-treated control group were performed using Kruskal–Wallis test followed by uncorrected Dunn’s test for multiple comparisons (if *p* < 0.05). *p* < 0.05 is considered statistically significant. Statistical analyses were performed with SPSS (IBM SPSS Statistics, version 24.0, Chicago, IL, USA), and scatter plots were created using GraphPad Prism version 7.0a (GraphPad Software; San Diego, CA).

## Results

**Fig. 2:**
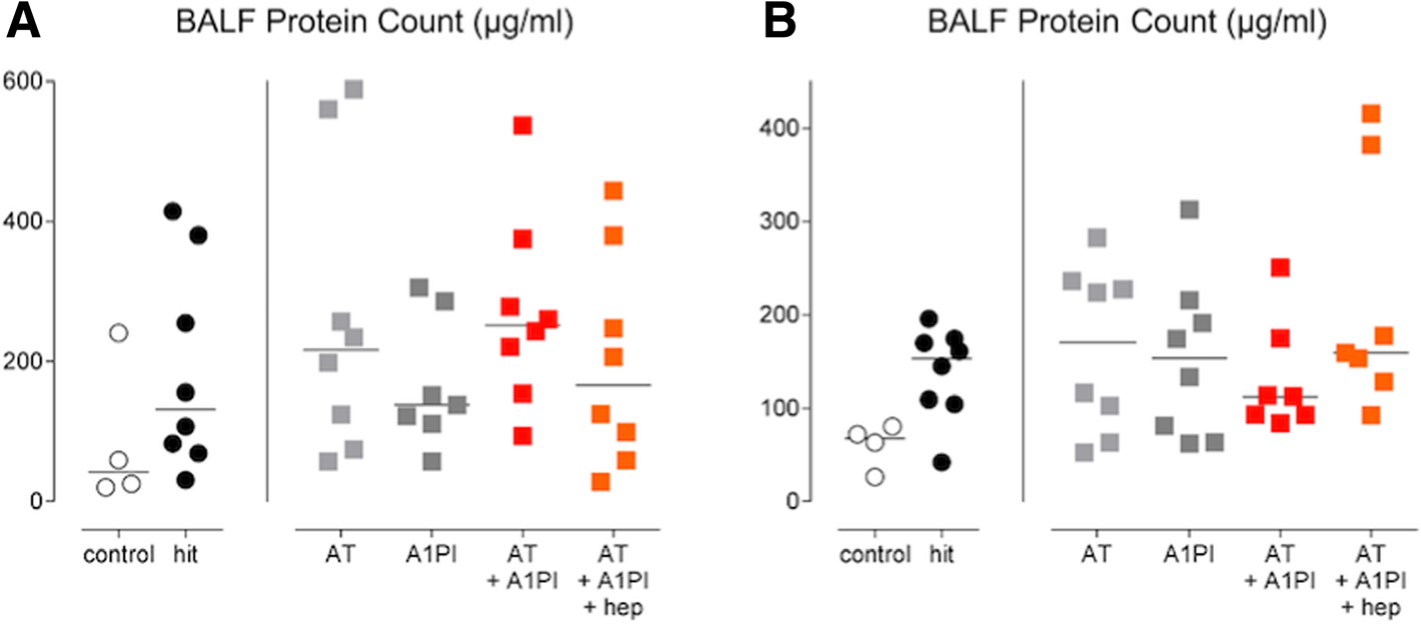
Marker for acute lung injury. Single-hit lung injury model: **a** protein count in BALF. Two-hit lung injury model: **b** protein count in BALF. Abbreviations: BALF, bronchoalveolar lavage fluid; AT, antithrombin; A1PI, alpha-1 protease inhibitor; Hep, heparin

**Fig. 3:**
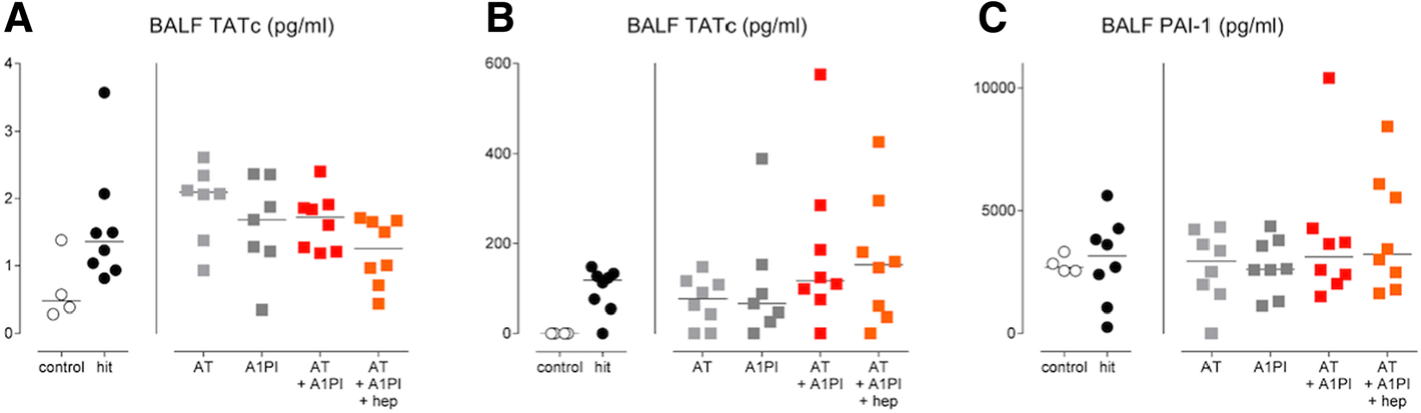
Markers of pulmonary coagulation. Single-hit lung injury model: **a** TATc levels in BALF. Two-hit lung injury model: **b** TATc levels in BALF. **c** PAI-1 levels in BALF. Abbreviations: BALF, bronchoalveolar lavage fluid; AT, antithrombin; A1PI, alpha-1 protease inhibitor; Hep, heparin; TATc, thrombin–antithrombin complexes; PAI-1, plasminogen activator inhibitor 1

**Fig. 4:**
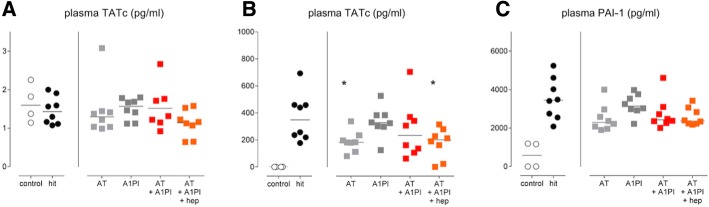
Markers of systemic coagulation. Single-hit lung injury model: **a** TATc levels in plasma. Two-hit lung injury model: **b** TATc levels in plasma. **c** PAI-1 levels in plasma. *p < 0.05. Abbreviations: AT, antithrombin; A1PI, alpha-1 protease inhibitor; Hep, heparin; TATc, thrombin–antithrombin complexes; PAI-1, plasminogen activator inhibitor 1

**Fig. 5:**
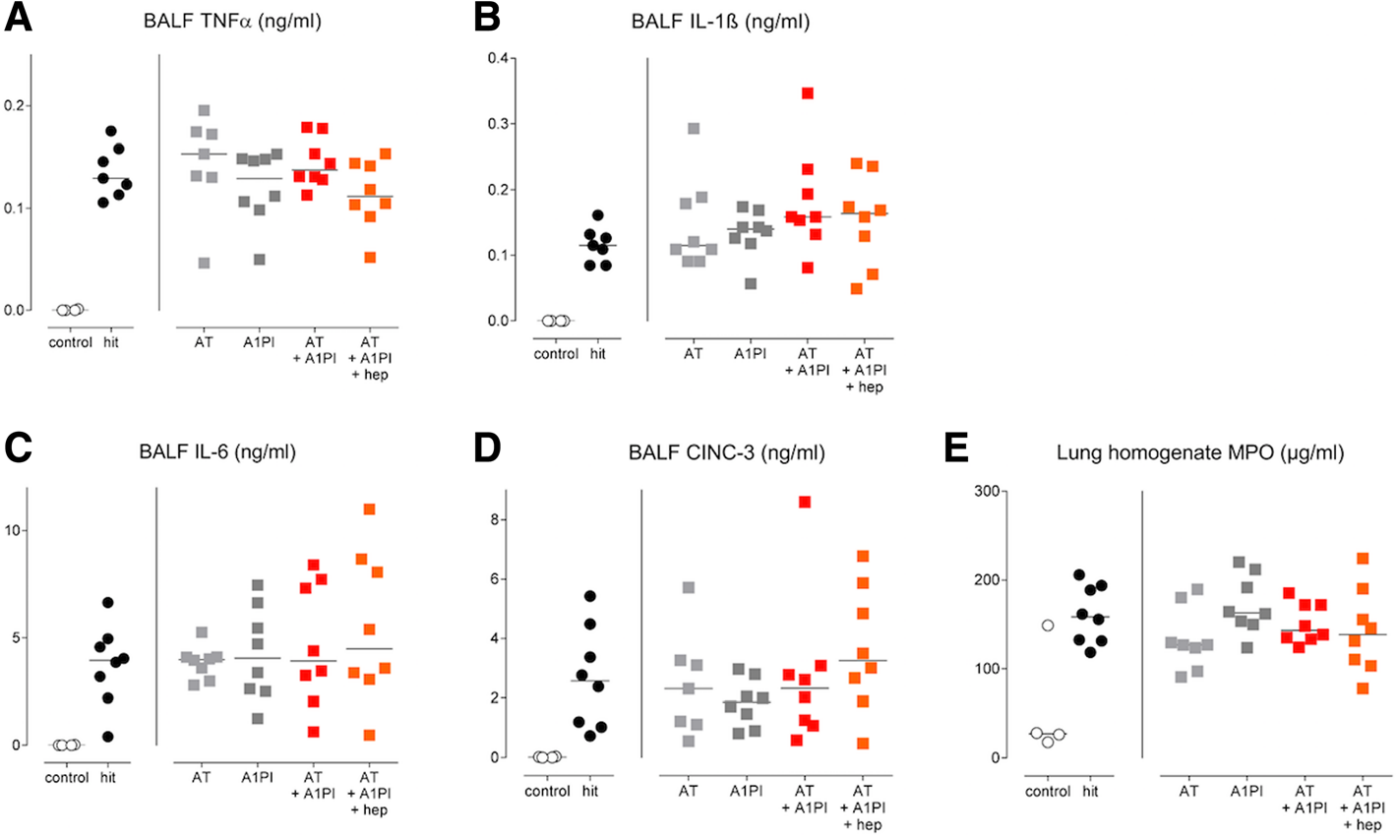
Markers of pulmonary inflammation. Single-hit lung injury model: **a** TNFα levels in BALF. **b** IL-1β levels in BALF. Two-hit lung injury model: **c** IL-6 levels in BALF. **d** CINC-3 levels in BALF. **e** MPO levels in lung homogenate. Abbreviations: AT, antithrombin; A1PI, alpha-1 protease inhibitor; BALF, bronchoalveolar lavage fluid; CINC, cytokine-induced neutrophil chemoattractant; Hep, heparin; IL, interleukin; MPO, myeloperoxidase; TNF, tumor necrosis factor

**Fig. 6:**
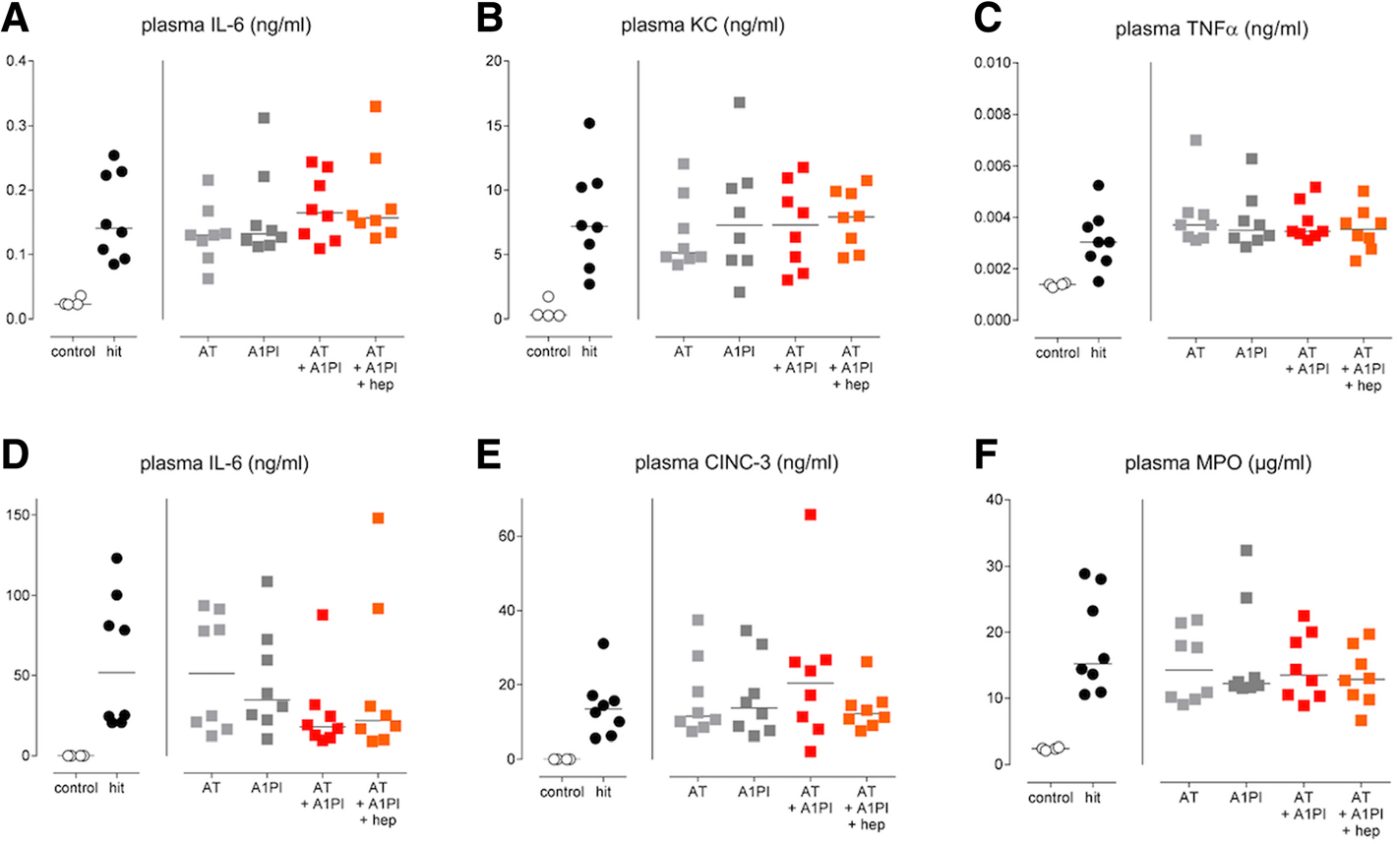
Markers of systemic inflammation. Single-hit lung injury model: **a** IL-6 levels in plasma. **b** KC levels in plasma. **c** TNFα levels in plasma. Two-hit lung injury model: **d** IL-6 levels in plasma. **e** CINC-3 levels in plasma. **f** MPO levels in plasma. Abbreviations: AT, antithrombin; A1PI, alpha-1 protease inhibitor; CINC, cytokine-induced neutrophil chemoattractant; Hep, heparin; IL, interleukin; KC, keratinocyte chemoattractant; MPO, myeloperoxidase; TNF, tumor necrosis factor

Lung injury was induced in both models evidenced by increased levels of protein in the BALF indicative of acute lung injury as well as coagulation and inflammatory parameters in the hit group compared to the control group (Figs. 2, 3, 4, 5, and 6). Notably, the two-hit lung injury model elicited a far more vigorous pulmonary coagulation and inflammatory response than the single-hit model.

No abnormal macroscopic bleeding was observed in any of the animals. Intraperitoneal administration of AT and A1PI resulted in increased levels of the agents within the pulmonary compartment (data not shown). In the single-hit lung injury model, all animals survived the experiment. In the two-hit lung injury model, all animals survived i.v. injection of LPS and the following 2 h without medical intervention. The two-hit lung injury model yielded a mortality of 2.5% (1 out of 40). It concerned one animal from the A1PI group with the terminal endpoint being prolonged hypotension (MAP < 55 mmHg) unresponsive to fluid resuscitation followed by severe lactate acidosis after 3 h of mechanical ventilation. The experiment was repeated to achieve a similar number of animals per group.

### Coagulation and fibrinolysis

Both lung injury models induced pulmonary coagulopathy as shown by elevated TATc levels in BALF (*p < 0.05 hit* versus *control*). Fibrinolysis, as depicted by PAI-1 levels, was not affected within the pulmonary compartment in the two-hit lung injury model (*p = 0.55 hit* versus *control*). None of the interventions exerted a significant effect on pulmonary coagulopathy in the models studied (Fig. 3).

Systemic coagulation was not enhanced in the single-hit lung injury model. In the two-hit lung injury model, systemic hypercoagulability and reduced fibrinolysis were present in all animals as proven by increased plasma levels of TATc and PAI-1 (*p < 0.01 hit* versus *control*, for both). Administration of AT significantly reduced systemic TATc levels (*p < 0.05 hit* versus *AT*) (Fig. 4).

### Inflammation and endothelial injury

In the single-hit lung injury model, i.n. LPS inoculation resulted in pulmonary inflammation shown by a marked increase of acute inflammatory markers TNFα and IL-1β in BALF (*p < 0.01 hit* versus *control*). Likewise, the two-hit lung injury model led to an acute inflammatory response within the lungs as shown by markedly elevated levels of IL-6, CINC-3, and MPO (*p < 0.01 hit* versus *control*, for all). None of the interventions affected the pulmonary inflammatory response (Fig. 5).

Both lung injury models also resulted in systemic inflammation as shown by markedly increased IL-6 and KC levels in plasma (*p < 0.01* versus *control*) in the single-hit lung injury model and elevated levels of IL-6, CINC-3, and MPO in plasma in the two-hit lung injury model (*p < 0.01 hit* versus *control*, for all). The interventions did not affect systemic inflammation (Fig. 6).

### Physiological parameters in the two-hit lung injury model

All animals showed a decrease in mean arterial pressure throughout the 4 h of mechanical ventilation, and 24% (11 out of 46) animals required additional fluid resuscitation. Those animals were evenly distributed between all groups, with only the AT group not having animals requiring fluid resuscitation. All animals showed a respiratory alkalosis mainly due to high tidal volume MV. Lactate levels were elevated in all animals confirming a model of septic shock. Inspiratory pressures ranged between 16 and 23 cmH_2_O, and a FiO2 of 30% was sufficient in all animals (Table 1).

**Table 1:** Physiological parameters of the two-hit lung injury model

Intervention group	MAP (mmHg)			pH			Lactate (mmol/L)			*P*_insp_ (mbar)		
	*T* = 2	*T* = 4	*T* = 6	*T* = 2	*T* = 4	*T* = 6	*T* = 2	*T* = 4	*T* = 6	*T* = 2	*T* = 4	*T* = 6
Hit	116	102	80	7.53	7.56	7.50	6.97	4.42	4.73	18	18	18
AT	112	92	77	7.57	7.62	7.59	6.05	4.61	4.65	20	20	21
A1PI	109	90	77	7.55	7.60	7.53	6.93	4.80	5.22	20	20	21
AT + A1PI	114	98	89	7.58	7.59	7.57	6.33	5.07	5.57	19	20	21
AT + A1PI + heparin	106	96	78	7.52	7.60	7.57	6.29	5.11	4.18	21	20	20

Abbreviations: *AT* antithrombin, *A1PI* alpha-1 protease inhibitor, *MAP* mean arterial pressure, *Pinsp* inspiratory pressure, *T* time point in h

## Discussion

The main results of this study are that neither single therapy with AT or A1PI nor combination therapy of both agents attenuates pulmonary coagulopathy or inflammation in two different murine models of acute lung injury.

Combination therapy of AT and A1PI does not exert a synergistic beneficial effect on pulmonary coagulation, nor on pulmonary inflammation. The only previous study investigating the combined effect of AT and A1PI showed that combination therapy prevents pathophysiological changes within the lungs [12]. The rationale of a synergistic effect of combination therapy comprises of two parts: AT is thought to reduce binding of neutrophils to the endothelium [21] leading to a decrease in neutrophil activation [22]. This in turn should result in lower levels of reactive oxygen species (ROS) from neutrophilic granules [23], known to inactivate A1PI [24, 25]. Both protease inhibitors were assumed to protect each other’s functionality and to exert a synergistic effect in their anticoagulant and anti-inflammatory capacity. Nonetheless, this present study does not support these assumptions.

Interestingly, this present experiment did not find a beneficial effect of AT on pulmonary coagulopathy and thereby contradicts previous studies administering the same dosage of AT [18, 19]. Differences in the applied animal models may attribute to the different outcomes as discussed below. This study confirms previous research [18, 19] that AT does not affect pulmonary or systemic inflammatory parameters in in vivo models [26].

In the Kybersept trial [27], an interaction between prophylactic heparin and AT was found. Therefore, we also studied the effect of adding heparin to the combination therapy. Co-administration of heparin did not result in an increased bleeding risk.

The current study assessed the effect of A1PI on pulmonary coagulation and inflammation for the first time and did not find an effect of A1PI on coagulopathy, nor on inflammation. An explanation may be the time frame of the experiment. A1PI is an acute-phase protein synthesized by the liver and seems to play a dual role within the inflammatory response [7]. A preclinical study sacrificing animals 2 h after A1PI administration found increased markers of inflammation [28], whereas most studies sacrificing animals at a later time point detected decreased inflammatory cytokines [20, 29, 30]. Hence, one may hypothesize that A1PI does exert its anti-inflammatory effects after the acute phase of lung injury but not in the early phase, as in our model.

This animal study has several strong points. First of all, this study investigated the effect of the interventions in two clinically relevant and validated models mimicking ARDS [31–34]. Secondly, rigorous methods such as randomization of animals, blinding of groups before analysis of outcomes, and a power calculation were used. Importantly, all interventions were administered as a treatment. Many previous preclinical studies chose to give AT or A1PI as a pre-treatment, hence before induction of lung injury—which is clinically less relevant [29, 30, 35, 36]. With regard to drug dosage, a dosage of 250 IU/kg has been proven to attenuate enhanced coagulation, but did not have a beneficial effect on inflammation in rat models [18, 19]. With regard to A1PI, the dosage used proved to be effective in previous murine models [20, 29]. It may be hypothesized that different etiologies of lung injury require different dosages and that higher dosages of AT or A1PI may exert a more pronounced effect.

One strength of this present study is actually also one of the major limitations. It is important to realize that both models are simulating lung injury within the acute phase, e.g., within the first 6 h after the induction of lung injury. Comparable studies are scarce, and most studies using lung injury models sacrificed animals after 16 up to 75 h focusing on a beneficial effect of therapy at a later time point [29, 30]. Hence, this study gives insight in the effects of drugs studied within the acute phase of lung injury, but does not allow to draw any conclusions about the effects at a later time point of lung injury.

In conclusion, in this present study, neither single therapy with AT or A1PI nor combination therapy exerts beneficial effects on pulmonary coagulation or inflammation in two murine acute lung injury models. AT markedly decreased systemic coagulopathy. Adding heparin did not result in aggravation of the anticoagulant effect leading to abnormal macroscopic bleeding. Further research is warranted to gain insights about the effects of AT and A1PI on coagulation and inflammation after the acute phase of lung injury.

## Abbreviations

A1PI: Alpha-1 protease inhibitor
ARDS: Acute respiratory distress syndrome
AT: Antithrombin
BALF: Bronchoalveolar lavage fluid
FiO_2_: Inspired oxygen fraction
i.n.: Intranasal
i.p.: Intraperitoneal
i.v.: Intravenous
IL: Interleukin
KDA: Ketamine, dexmedetomidine, atropine
LPS: Lipopolysaccharide
MPO: Myeloperoxidase
NaCl: Sodium chloride
PEEP: Positive end-expiratory pressure
SD: Standard deviation
TNF: Tumor necrosis factor
VILI: Ventilator-induced lung injury
